# Research of the Methylation Status of miR-124a Gene Promoter among Rheumatoid Arthritis Patients

**DOI:** 10.1155/2013/524204

**Published:** 2013-10-10

**Authors:** Qiao Zhou, Li Long, Guixiu Shi, Jing Zhang, Tong Wu, Bin Zhou

**Affiliations:** ^1^Department of Rheumatology, Sichuan Academy of Medical Science & Sichuan Provincial People's Hospital, No. 32 West Section 2, Yihuan Road, Chengdu, Sichuan 610072, China; ^2^Department of Rheumatology, The First Affiliated Hospital of Xiamen University, Xiamen, Fujian 361003, China

## Abstract

*Objective*. To analyze the methylation status of miR-124a loci in synovial tissues of rheumatoid arthritis (RA) patients using methylation-specific polymerase chain reaction (MSP). *Materials and Methods*. DNA obtained from the frozen tissue of 7 RA samples, 6 osteoarthritis (OA) samples, and 3 healthy controls were undergoing bisulfite conversion and then analyzed for miR-124a promoter methylation using MSP assay. *Results*. miR-124-a1 and miR-124-a2 promoter methylation were both seen in 71.4% of RA samples compared to 16.7% of OA samples. miR-124-a3 promoter methylation was seen in 57.1% of RA samples and 0% of OA samples. All the three loci were unmethylated in 3 healthy controls. *Conclusion*. The methylation status of miR-124a seen in this study concurs with that reported in tumor cells, indicating epigenetic dysregulation constituents, a mechanism in the development of rheumatoid arthritis.

## 1. Introduction

Rheumatoid arthritis (RA) is a chronic, inflammatory, symmetrical polyarticular autoimmune disease affecting ~1% of the population worldwide [1, 2]. The main characteristic features of RA are persistent inflammation, synovium hyperplasia, lymphocyte infiltration, and abnormal proliferation of fibroblast-like synoviocytes (FLS), which eventually lead to progressive cartilage erosion and bone destruction [3]. Although the pathogenesis of RA is not clear, much evidence demonstrates that microRNAs (miRNAs) display important roles in immune response [4, 5]. miRNAs are endogenous, short (about 19–25 nucleotides long) single-stranded, and noncoding RNAs that can influence the target mRNA processing at the posttranscriptional level [6] and play important roles in cell processes such as proliferation, apoptosis, differentiation, or even in tumorigenesis [7]. One of these important miRNAs is miR-124a. Accumulating evidence shows that miR-124a is downregulated in synovial tissues of RA patients compared with that of osteoarthritis (OA) patients [8]. For example, Nakamachi et al. found that its level in RA FLS was less than one-sixth of that seen in OA FLS [9]. However, the mechanism of the aberrant expression of miR-124a in RA synovial tissues is still unknown. It is found that miR-124a has three genomic loci (miR-124a-1 (8p23.1), miR-124a-2 (8q12.3), and miR-124a-3 (20q13.33)) that encode for the same mature miRNA. Interestingly, the miR-124a-1 and miR-124a-3 genes are located within CpG islands, whereas miR-124a-2 is 760 bp downstream of a CpG island. Studies in several cancer cells demonstrated that all the three loci are silenced by the hypermethylation of its promoter [10, 11]. Agirre et al. pointed out that the corresponding CpG islands of miR-124a-1 and miR-124a-3 are frequently methylated in acute myeloid leukemia [12]. Since reported data showed that the expression of miR-124a was suppressed in RA synovial tissues, it would be of interest to investigate whether epigenetic mechanism, especially DNA methylation, has played a role in it.

## 2. Materials and Methods 

### 2.1. Synovial Samples

Synovial tissues were obtained from RA patients, OA patients, and joint trauma patients (healthy control specimens) undergoing total knee arthroplasty from October 2012 to April 2013 in Sichuan Provincial People's Hospital. Tissue was snap-frozen and stored at −80°C. RA and OA were diagnosed according to the criteria of the American College of Rheumatology [13, 14]. The clinical characteristics of the patients are shown in Table 1. Samples were obtained in accordance with the Declaration of Helsinki Ethical Principles for Medical Research Involving Human Subjects, as approved by the World Medical Association. All patients signed informed consent forms, and the study was approved by the Ethics Committees of Sichuan Provincial People's Hospital.

### 2.2. DNA Extraction and Bisulfite Conversion

 Genome DNA was extracted from 25 mg of frozen synovial tissue using the PureLink Genomic DNA mini kit (Invitrogen, USA). DNA was quantitated using the Nanodrop (Nanodrop technologies, USA). 2 *μ*g DNA was used for bisulfite conversion as described [15]. Modified DNA was purified using the QIAEX II Gel Extraction kit (QIAGEN, Germany) according to the manufacturer.

### 2.3. Methylation-Specific PCR

The DNA methylation status was analyzed by methylation-specific PCR (MSP) using primers specific for either the methylated or bisulfate modified unmethylated DNA. 1.5 *μ*L bisulfite-converted DNA was amplified using 0.15 *μ*L primers (Sangon Biotech, Shanghai), 0.45 *μ*L MgCl_2_ (25 mM), 1.8 *μ*L dNTP (2.5 mM each), 6.75 *μ*L 2x GC buffer (I) (Takara, Japan), and 1.6 *μ*L* Takara LA taq* polymerase (Takara, Japan). Each step included methylated (RKO, ATCC CRL-2577) and unmethylated (MGC-803, ATCC) controls along with nontemplate control. The amplified products were run on a 2% agarose gel with an expected size of 164, 185, and 150 bp (three loci each) for a methylated product and 165, 180, and 155 bp (three loci each) for an unmethylated product.

### 2.4. Statistical Analysis

Statistical comparisons between groups were carried out by Chi square test or Fisher's exact test. *P* values less than 0.05 were considered significant. 

## 3. Results

### 3.1. General Clinical Features

The mean age of the 7 RA patients was 63 (range 58–72) years, with a female to male ratio of 2.5 : 1. The mean presurgical CRP and ESR were 2.4 ± 1.5 mg/dL and 14 ± 9 mm/h, respectively. The mean DAS28 was 1.86 ± 0.57. The three healthy control patients underwent surgery because of car accident, falling off, and fighting, respectively.

### 3.2. Methylation Status of miR-124a

**Figure 1:**
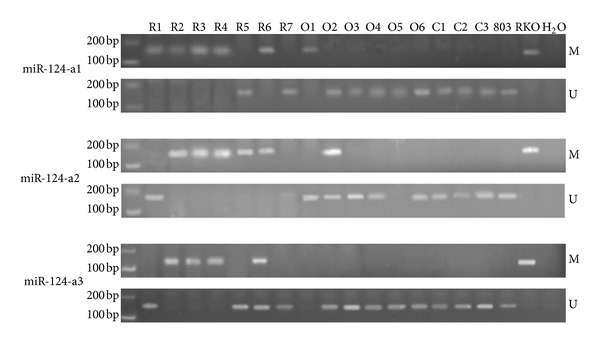
Gel electrophoresis of the MSP products for miR-124-a1, miR-124-a2, and miR-124-a3 in RA, OA patients, and healthy controls. R: rheumatoid arthritis; O: osteoarthritis; C: healthy control; 803 is unmethylated positive control; RKO is methylated positive control; H_2_O is blank control.

The methylation status of miR-124a was evaluated in 7 RA, 6 OA, and 3 control synovial tissues (Figure 1). All of the RA tissues were hypermethylated: 5 for miR-124a-1 (5/7), 5 for miR-124a-2 (5/7), and 4 for miR-124a-3 (4/7). Among them, 4 tissues (4/7) showed promoter methylation of all three gene loci, and the frequency was 57.1%, higher than that of OA (0, 0/6) and control (0, 0/3) (Table 2). The methylation frequency of the three genes in RA synovial tissues has no statistical significance compared to each other.

## 4. Discussion

Rheumatoid arthritis is one of the common autoimmune diseases, and the molecular mechanism of its pathogenesis is unclear now. miRNAs have been implicated in the pathogenesis of malignant and nonmalignant disease. The microRNA miR-124a was initially identified as a crucial regulator involved in neurogenesis [16]. In neuronal tissues, miR-124a contributes to the differentiation of neural progenitors into mature neurons [17]. Pierson et al. reported that the 3′-UTR of CDK-6 mRNA is a direct target of miR-124 and that CDK-6 expression is suppressed by miR-124 overexpression in medulloblastoma cell lines [18]. Nakamachi et al. confirmed that the expression of CDK-6 protein was higher in RA FLS than in OA FLS and that CDK-6 expression was suppressed when pre-miR-124a was transfected into RA FLS; thus, the cell cycle was arrested at the G1 phase [9]. Since CDK-6 is known as a G1/S phase regulator, it is speculated that miR-124a is an important regulator of the G1/S transition in synovial tissue as well as in tumors. 

We decided to focus on the methylation status of miR-124a in RA synovial tissues for several reasons. (a) The expression of miR-124a is downregulated in RA synovial tissues. (b) Hypermethylation of gene promoters is a frequent mechanism of gene silencing. (c) The three loci of miR-124a are either located within a CpG island or somewhere downstream a CpG island. (d) It is reported that miR-124a has been silenced by gene promoter hypermethylation in several cancer cell lines. The results of our study support the approach, showing that the gene promoter of miR-124a is hypermethylated, and this might associate with its downregulation in RA synovial tissues.

 The methylation frequencies of the three gene loci observed among RA synovial tissues (57.1%~71.4%) were much higher than that observed among OA (0~16.7%) and control synovial tissues (0). However, the *P* values were not so significant considering the small sample size. 

We obtained good-quality DNA from all frozen tissues, yet there were some RA samples that showed both methylated and unmethylated band. We considered the following reasons: (a) although we carefully removed all connective tissue and fat, the area chosen might still have very little nonsynovial tissue to confound the results. It would be pertinent to point out that small amounts of contaminating nonsynovial tissues could become a source of contamination and could provide false negative results. This issue emphasizes the need to carefully choose samples for detection of methylation status, therefore avoiding potential confounders. (b) Regions within the synovial tissue might be variably methylated, even within the individual clones of the same tissue sample, and the synovial tissue could be a mosaic of variable degrees of methylation. Thus we speculate mosaicism could have contributed to our findings here. This issue could be solved by bisulfite-sequencing.

This study highlights the methylation pattern of RA synovial tissues compared with that of OA and healthy control. Although it has some limitations such as the number of samples analyzed, the study still provides useful information on promoter methylation pattern of miR-124a. This epigenetic dysregulation of miR-124a in RA synovial tissue constitutes an emerging mechanism implicated in the development of RA and will provide us with new excellent targets for DNA demethylating agents.

## Figures and Tables

**Table 1:** Clinical characteristics of the patients*.

Patient	Age	Sex	Disease duration (years)	Presurgical CRP (mg/dL)	Presurgical ESR (mm/h)	DAS 28	Medications
RA1	72	F	17	1.9	17	2.42	MTX 10 mg qw LEF 20 mg qd
RA2	58	F	9	1.5	21	2.16	MTX 12.5 mg qw LEF 10 mg qd
RA3	63	M	21	2.7	13	1.87	Pred. 7.5 mg qd MTX 12.5 mg qw
RA4	65	F	15	<1	2	1.21	Pred. 5 mg qd LEF 20 mg qd
RA5	59	M	6	3.2	5	1.49	Pred. 5 mg qod MTX 10 mg qw
RA6	66	F	8	<1	9	1.23	LEF 20 mg qd
RA7	61	F	13	5.3	28	2.62	MTX 15 mg qw
OA1	72	F	—	<1	3	—	—
OA2	75	M	—	<1	2	—	—
OA3	69	M	—	<1	4	—	—
OA4	74	M	—	3.3	9	—	—
OA5	68	F	—	<1	3	—	—
OA6	70	M	—	2.8	11	—	—
C1	39	M	—				
C2	45	M	—				
C3	43	F	—				

*RA: rheumatoid arthritis; OA: osteoarthritis; CRP: C-reactive protein; ESR: erythrocyte sedimentation rate; DAS: disease activity score; MTX: methotrexate; Pred.: prednisone; LEF: leflunomides; C: control.

**Table 2:** Methylation status of miR-124a in synovial tissues of RA and OA*.

Gene	Methylation frequency			*P* value	
	RA	OA	C	RA versus OA	RA versus C
miR-124-a1	71.4%	16.7%	0	0.07	0.08
miR-124-a2	71.4%	16.7%	0	0.07	0.08
miR-124-a3	57.1%	0	0	0.05	0.17
All three loci	57.1%	0	0	0.05	0.17

*RA: rheumatoid arthritis; OA: osteoarthritis; C: healthy control.
